# Development of a Point-of-Care Ultrasound Track for Internal Medicine Residents

**DOI:** 10.1007/s11606-022-07505-5

**Published:** 2022-06-17

**Authors:** Robert Nathanson, Minh-Phuong T. Le, Kevin C. Proud, Charles M. LoPresti, Elizabeth K. Haro, Michael J. Mader, Jane O’Rorke, Patricia I. Wathen, Nilam J. Soni

**Affiliations:** 1 Medicine Service, South Texas Veterans Health Care System, San Antonio, TX USA; 2 Division of General & Hospital Medicine, Department of Medicine, UT Health San Antonio, San Antonio, TX USA; 3 Division of Pulmonary & Critical Care, Department of Medicine, UT Health San Antonio, San Antonio, TX USA; 4 Hospital Medicine Service, University Hospitals, Cleveland, OH USA; 5 Department of Medicine, Case Western Reserve University School of Medicine, Cleveland, OH USA

**Keywords:** education, point of care, retention, ultrasound

## Abstract

**Background:**

Point-of-care ultrasound (POCUS) training has been increasing among internal medicine (IM) residency programs, but few programs can provide longitudinal training due to barriers such as lack of trained faculty.

**Aim:**

Describe the development of a longitudinal POCUS track for IM residents using local and external resources, including a national POCUS certificate program.

**Setting:**

University-based IM residency program affiliated with a public and veterans affairs hospital.

**Participants:**

Twelve IM residents from 2018 to 2021.

**Program Description:**

Residents complete a national POCUS certificate program by attending live courses and completing online modules, an image portfolio, and final knowledge/skills assessments. Locally, residents participate in 1-month procedure and diagnostic POCUS rotations and provide peer-to-peer POCUS teaching of residents and medical students.

**Program Evaluation:**

The POCUS track increased residents’ use and comfort with diagnostic and procedural applications. All residents rated being satisfied or very satisfied with the track and would recommend it to prospective applicants (100%). The most commonly reported barriers to utilizing POCUS per residents were time constraints (83%), lack of available ultrasound equipment (83%), and lack of trained faculty (58%).

**Discussion:**

IM residency programs with limited faculty expertise in POCUS can leverage external resources to provide longitudinal POCUS training to its residents.

**Supplementary Information:**

The online version contains supplementary material available at 10.1007/s11606-022-07505-5.

## INTRODUCTION

Internal medicine (IM) physicians are increasingly incorporating point-of-care ultrasound (POCUS) into patient care for procedural and diagnostic applications.^1^ POCUS use to guide bedside procedures improves patient safety by increasing procedural success rates, reducing complications, and avoiding unnecessary attempts and has evolved to become the standard of care for certain bedside procedures.^2–7^ Diagnostic POCUS applications can improve diagnostic accuracy, prognostication, patient satisfaction, and shared diagnostic understanding.^6, 8, 9^ By guiding clinical decision-making, POCUS can contribute to more efficient and cost-effective medical care.^10^

The Alliance for Academic Internal Medicine (AAIM) has endorsed POCUS training in IM residency programs, and programs have been seeking creative ways to implement POCUS training.^11^ Despite the high demand^12–14^, incorporation of POCUS curricula by IM residency programs has been slow, increasing from 25% to 37.5% between 2012 and 2016.^15, 16^ Major barriers to incorporating POCUS training in IM residency programs include lack of faculty with POCUS expertise, time and cost of training faculty, and time required to train residents.^15–19^

To meet the demand for POCUS training, some IM residency programs have created POCUS electives or held workshops that provide an immersive experience.^13, 18, 20–22^ However, longitudinal POCUS training has been shown to increase knowledge and skills retention among IM residents better than stand-alone workshops, but few programs currently can offer longitudinal POCUS training.^23, 24^ Among the IM residency tracks in hospital medicine in 2017, only one included a rotation in ultrasound diagnostics, and none was dedicated to POCUS training.^25^

In 2018, we created a unique IM residency track, the POCUS track, that utilizes both local and external resources through a national certificate program in order to provide a 3-year longitudinal POCUS training experience. Here, we describe the development of our POCUS residency track including resources required, perspectives of residents and residency program leadership, and barriers to establishing a POCUS track.

## SETTING AND PARTICIPANTS

We developed a POCUS track for our IM residency, a university-based program with 95 categorical residents that is affiliated with a public and veterans affairs hospital. Candidates apply for the POCUS track through a separate NRMP number. Our program accepts 4 residents per year with a total of 12 residents in all three years on the POCUS track.

## PROGRAM DESCRIPTION

POCUS track residents receive longitudinal ultrasound training during all three years (Supplemental Table 1).

### Year 1

#### Online Modules

Interns complete self-directed online modules on the fundamentals of ultrasound and focused cardiac, pulmonary, and vascular ultrasound per the Society of Hospital Medicine – American College of Chest Physicians (SHM-ACCP) POCUS Certificate of Completion (COC) program.^26^ Interns are recommended to complete the modules during an elective rotation as the modules require 10–20 h of work.

#### Procedure Rotation

All IM interns participate in a 1-month procedure service rotation focusing on ultrasound-guided paracentesis, thoracentesis, and lumbar puncture. Large joint arthrocentesis and vascular access procedures are occasionally performed. Procedures are performed by interns under the supervision of a procedure chief resident or attending hospitalist.

#### POCUS CME Course #1

Interns attend an introductory 2- or 3-day POCUS course per the SHM-ACCP POCUS COC program that teaches goal-directed echocardiography, pulmonary, vascular, and abdominal ultrasound applications through a combination of lectures, image interpretation sessions, and hands-on scanning sessions with live models. Afterwards, interns are encouraged to start collecting images for their portfolios.

### Year 2

#### Image Portfolio

Residents are registered for the SHM-ACCP POCUS COC program’s image portfolio in year 1 and focus on building their portfolios in year 2. The COC program requires 209 images of the heart, lungs, abdomen, and lower extremity vasculature. Expert SHM POCUS faculty use standardized image quality criteria to provide feedback on the images. Residents largely collect portfolio images during their inpatient rotations and POCUS elective.

#### POCUS Elective

During this 4-week rotation, residents receive refresher training on focused cardiac, pulmonary, lower extremity vascular, abdominal, and other POCUS applications, including clinical integration of findings into the management of shock and cardiac arrest. Training is led by the POCUS track faculty director and includes a combination of didactics and hands-on scanning sessions in our Center for Clinical Ultrasound Education. During this rotation, residents attend four 2-h scanning sessions for image acquisition practice with live models, continue building their image portfolios, and meet weekly to bi-weekly with the POCUS track faculty director to review their collected images.

#### POCUS CME Course #2

SHM-ACCP POCUS COC participants must attend an approved regional POCUS course. Currently, approved courses are offered in San Francisco, Denver, New York, Minneapolis, Chicago, and San Antonio. In addition to reviewing core POCUS applications, these courses provide training in ultrasound-guided procedures; basic skin, soft tissue, and joint ultrasound; and discussion of ultrasound program development.

### Year 3

#### POCUS Teaching

Third-year POCUS track residents are assigned POCUS teaching activities to solidify their knowledge and skills and help meet the demand for POCUS training of residents and medical students. Our residency program utilizes a 4+1 block schedule, and one half-day is dedicated to POCUS teaching during each ambulatory week. Peer-to-peer instruction is provided during skills workshops and hands-on scanning sessions of the resident POCUS elective and during various medical student POCUS sessions. Additionally, the POCUS track residents themselves created and implemented new POCUS workshops for our IM residency program.

#### Final Assessments

The final knowledge and skills exams are taken in the spring of the 3^rd^ year of IM residency during the SHM annual conference or an approved regional POCUS course. During the COVID-19 pandemic, both the written and skills exams were administered virtually in 2020–2021. The written exam was conducted online through the SHM learning portal and proctored by SHM staff virtually. The skills exam was administered virtually by an expert SHM POCUS faculty using REACTS tele-ultrasound software (Philips/Innovative Imaging Technologies, Montreal, Canada). After passing the final knowledge and skills exams, POCUS track residents were granted a certificate for completing the SHM-ACCP POCUS program.

### Resources Required

Resources for developing our POCUS track are summarized in Table 1. The POCUS track faculty director serves as a clinical POCUS mentor and supervisor of residents, liaison to the SHM-ACCP POCUS COC program, and collaborator with the IM residency program leadership. Other specific responsibilities include leading the POCUS elective, coordinating the ambulatory-week POCUS teaching activities, and providing instruction and feedback on peer-to-peer ultrasound teaching. The POCUS track faculty director role requires at least 15% protected time.

**Table 1:** Resources Required for a 3-Year POCUS Track

Resource	Details
SHM-ACCP POCUS Certificate	• Approximate total cost per resident = ~$7000 ○ SHM resident membership = $300 ○ Online modules = $500 ○ 2 POCUS CME courses = $3200 ○ Travel = $1,500* ○ Image portfolio= $1400 ○ Final Assessment Fee= $100
POCUS Track Faculty Director	• Faculty with POCUS expertise (~15% FTE) • Responsibilities: ○ Coordinate POCUS teaching activities ○ Serve as mentor and advisor to track residents ○ Serve as liaison to SHM-ACCP POCUS COC Program ○ Teach POCUS elective
Ultrasound Equipment	• Ultrasound machine(s) ○ Cart-based system ($20–50K) ○ Handheld device ($4–10K) • Transducers† ○ Linear-array ○ Phased-array • Image transfer capabilities
Procedure Rotation & POCUS Elective Rotation	• Faculty director ○ Protected time commensurate on hours per month dedicated to rotation (~10% FTE per full-time month dedicated to rotation) • Supplies ○ Live models from medical school standardized patient pool (~$20-30/hour per model for hands-on scanning practice) ○ Procedure task trainers (~$15K for 1 set of paracentesis, thoracentesis, central line, lumbar puncture) ○ Ultrasound machines (either dedicated or shared)
Administrative Support	• Program Coordinator in IM Residency Program (~5–10% FTE) • Responsibilities: ○ Course registration, reimbursement processing, coordinating schedules of POCUS track residents, and supporting POCUS track faculty director

*Based on shared occupancy of 2 residents per hotel room

†Need linear and phased-array transducers at minimum but having a curvilinear transducer can be advantageous for some applications

*POCUS*, point of care ultrasound; *SHM*, Society of Hospital Medicine; *ACCP*, American College of Chest Physicians; *FTE*, full-time equivalent; *COC*, Certificate of Completion; *IM*, internal medicine

Completing a training certificate through the SHM-ACCP POCUS COC program costs approximately $7000 per resident including travel. Our IM residency program had to be creative about securing institutional funding for this program. At our institution, university funds were reallocated to accept up to 4 residents/year on the POCUS track. We were fortunate that our Department of Medicine leadership supported the creation of the POCUS track.

## PROGRAM EVALUATION

All POCUS track residents were surveyed in April 2021 with a response rate of 100% (Table 2). The project was reviewed by the IRB and deemed to be non-research. Characteristics of POCUS track residents are summarized in Supplemental Table 2.

**Table 2:** Resident Feedback from End-of-Year Survey of POCUS Track

General POCUS Track Feedback (*n*=12)	*n* (%)
Overall satisfaction with the POCUS track	
*Very dissatisfied, Dissatisfied, or Neutral* *Satisfied* *Very satisfied*	0 (0) 7 (58) 5 (42)
Satisfaction with SHM-ACCP POCUS COC Program	
*Very dissatisfied or Dissatisfied* *Neutral* *Satisfied* *Very satisfied*	0 (0) 2 (17) 7 (58) 3 (25)
Recommend POCUS track to prospective applicants	
*Yes* *No*	12 (100) 0 (0)
POCUS skills learned on the POCUS track will help in my specialty and future career	
*Strongly disagree or Disagree* *Neutral* *Agree* *Strongly agree*	0 (0) 1 (8) 3 (25) 8 (67)
Participation in the POCUS track creates unique scholarship opportunities for residents (e.g., preparing lectures, posters, abstracts, manuscripts)	
*Strongly disagree or Disagree* *Neutral* *Agree* *Strongly agree*	0 (0) 2 (17) 5 (42) 5 (42)
POCUS elective (*n*=8)	
Satisfaction with POCUS elective	
*Very dissatisfied, Dissatisfied, or Neutral* *Satisfied* *Very satisfied*	0 (0) 2 (25) 6 (75)
Peer-to-peer POCUS teaching experience (*n*=8)	
Satisfaction with ambulatory-week POCUS teaching experience	
*Very dissatisfied, Dissatisfied, or Neutral* *Satisfied* *Very satisfied*	0 (0) 1 (12) 7 (88)
I enjoy teaching my colleagues and other clinicians about POCUS	
*Strongly disagree, Disagree, or Neutral* *Agree* *Strongly agree*	0 (0) 1 (12) 7 (88)
Senior resident feedback	
Because of the POCUS track, I am more likely to use POCUS after I complete residency (*n*=8)	
*Strongly disagree, Disagree, or Neutral* *Agree* *Strongly agree*	0 (0) 2 (25) 6 (75)
Completing the SHM-ACCP POCUS COC was advantageous for my job search or fellowship application (*n*=4)	
*Strongly disagree, Disagree, or Neutral* *Agree* *Strongly agree*	0 (0) 2 (50) 2 (50)
Participation in the POCUS track creates unique teaching opportunities for residents (*n*=4)	
*Strongly disagree, Disagree, Neutral, or Agree* *Strongly agree*	0 (0) 4 (100)
Barriers to POCUS use	
No barriers	0 (0)
Time constraints	10 (83)
Lack of available ultrasound equipment	10 (83)
Not enough faculty	6 (50)
Lack of comfort with scanning independently without supervision	2 (17)
Difficulty finding agreeable patients to practice scanning	1 (8)
Other	0 (0)

*POCUS*, Point of care ultrasound; *SHM*, Society of Hospital Medicine; *ACCP*, American College of Chest Physicians; *COC*, Certificate of Completion

Frequently reported reasons for choosing the POCUS track included the desire to obtain POCUS training and certification, teaching opportunities, and important skills for career development. All residents rated being satisfied or very satisfied overall with the POCUS track and would recommend it to prospective applicants. All third-year POCUS track residents successfully completed the SHM-ACCP POCUS COC program prior to graduation and felt participation in the track was advantageous for their job search or fellowship application. The most commonly reported barriers to utilizing POCUS per POCUS track residents were time constraints (83%), lack of available ultrasound equipment (83%), and too few faculty trained in POCUS to supervise scanning (58%).

Frequency of use and comfort levels for different diagnostic and procedural POCUS applications are shown in Supplemental Tables 3 and 4. In general, the reported frequency of use and comfort levels increased between the 1^st^ and 3^rd^ years of residency.

## DISCUSSION

We have described the development of an IM residency POCUS track combining local and external educational resources to provide longitudinal POCUS training. Direct benefits to POCUS track residents included the attainment of POCUS knowledge and skills and completion of a certificate program. Indirect benefits included increased institutional capacity for POCUS training by creating additional POCUS instructors for peer-to-peer teaching of residents and medical students. Our experience revealed important barriers and challenges that can help an IM residency program interested in developing a POCUS track.

POCUS skills are highly desired but inconsistently taught in IM residency programs across the country.^15, 16^ Longitudinal POCUS training has been shown to increase the frequency of ultrasound use and increase retention of knowledge and skills.^11, 18, 23, 24^ Creation of a POCUS track allowed residents with a deep interest in POCUS to receive comprehensive, longitudinal training and complete a certification endorsed by two national specialty organizations. POCUS track residents’ comfort and frequency of POCUS use increased and overall satisfaction with the program was positive. However, we recognize that our total sample size is relatively small, and future surveys will give us a better understanding of residents’ comfort level with different POCUS applications.

Several institutional benefits were realized by the creation of a POCUS track for IM residents. First, the lack of POCUS-trained IM faculty is a major barrier to POCUS implementation nationwide.^15, 18, 19^ For programs with limited local expertise, our POCUS track can serve as a model for providing longitudinal training by leveraging available external educational resources. We utilized the SHM-ACCP POCUS COC program, a nationally recognized POCUS certificate program, to overcome a shortage of local POCUS-trained faculty to provide feedback and assess the knowledge and skills of our POCUS track residents. Second, peer-to-peer instruction has been shown to be effective for POCUS education.^27–29^ POCUS track residents increased our institutional capacity to provide POCUS training by serving as instructors to teach residents and medical students. Third, our residency program anticipated attracting competitive candidates due to the uniqueness of the POCUS track. The number of candidates matching the POCUS track coming from the first quartile of our residency program’s rank list has been increasing since 2019. Fourth, POCUS track residents have increased the residency program’s scholarly output, including peer-reviewed publications and national conference presentations. Additionally, a POCUS track can provide early faculty development during residency and may better prepare residents for careers in academic medicine.^30^ Thus far, one-third of our POCUS track residents have been recruited as academic hospitalist faculty or chief residents.

The most commonly reported barriers to POCUS use per POCUS track residents were time constraints, lack of available ultrasound equipment, and limited number of faculty trained in POCUS to supervise scanning. These barriers are consistent with past national surveys of POCUS training in IM residency programs.^15, 16^ Specific challenges per POCUS track residents were completion of the image portfolio (limited protected time to collect images; limited availability of faculty to review and provide feedback on images) and arranging schedules to attend required in-person courses. Lack of available ultrasound equipment presented challenges for both portfolio development and skills practice, and our IM residency program recently purchased two handheld ultrasound units specifically for the POCUS track residents to overcome this barrier.

We recognize our experience has limitations. First, core components of our POCUS track have been demonstrated to increase knowledge and skills of practicing clinicians, including participation in 2- or 3-day immersive POCUS CME courses^31, 32^ and collection of an image portfolio.^33^ However, the impact of a POCUS track on IM residents’ long-term knowledge and skills retention, and changes to clinical practice is unknown. Furthermore, the current training paradigm provides limited experience in clinical integration of POCUS findings into bedside decision-making, but as more faculty become trained, residents will have more frequent supervised clinical integration in the future. Second, peer-to-peer POCUS instruction has been shown to be effective for medical student POCUS training,^27–29^ but its effectiveness among IM residents has not been well studied which we plan to evaluate in the coming years. Third, POCUS use is beneficial in outpatient settings to monitor high-risk patients for decompensation, expedite workups, and improve the availability of diagnostic resources for underserved populations.^34^ Our POCUS track curriculum focuses primarily on inpatient applications, and additional training in outpatient applications, including skin, soft tissues, and joint ultrasound, shall be added to the curriculum in the future. Finally, the costs of the national certificate program and availability of a local POCUS faculty director may be limitations for residency programs desiring to start a POCUS track. Alternatively, institutions without local expertise could support interested faculty in completing the SHM-ACCP POCUS COC program. Investing in the development of institutional POCUS faculty champions could allow for the creation of a local certificate program for IM residents, similar to the SHM-ACCP POCUS COC program.

In conclusion, we have described the development of a dedicated POCUS track in IM residency that can provide longitudinal POCUS training and certification for a select group of IM residents. Our POCUS track leverages external educational resources to help overcome local barriers to POCUS training for IM residents. Our POCUS track may serve as a model for IM residencies interested in providing longitudinal training to its residents but lack the required resources or local expertise to offer such training.

## Supplementary Information

ESM 1 (DOCX 47 kb)
