# Supplementary material for: Development of a Point-of-Care Ultrasound Track for Internal Medicine Residents
Source: J Gen Intern Med. 2022 Jun 17;37(9):2308–13. doi: 10.1007/s11606-022-07505-5 (PMC9205286; doi:10.1007/s11606-022-07505-5)
Supplement: Supplementary file 1 — (DOCX 47 kb) [file 11606_2022_7505_MOESM1_ESM.docx]

**Supplemental Table 1 – Curricular Components of a 3-year POCUS Track during Internal Medicine Residency**

| **Core Components** | | |
| --- | --- | --- |
| **Year 1** | **Year 2** | **Year 3** |
| - Online Modules - Procedure Rotation - POCUS CME Course #1 - Begin Image Portfolio | - POCUS Elective - POCUS CME Course #2 - Continue Image Portfolio | - Resident POCUS Teaching - UME POCUS Teaching - Finish Image Portfolio - Final Knowledge & Skills Exams |
| **Supplemental Components** | | |
| - Directed readings of book chapters and journal articles - Practice image acquisition and interpretation during inpatient rotations - Review questions and cases online | | |

POCUS, point of care ultrasound; CME, continuing medical education; UME, undergraduate medical education.

**Supplemental Table 2 – Characteristics of POCUS Track Residents**

| **Characteristic** | **n (%)** |
| --- | --- |
| Age, year | 29 (±2)* |
| Gender  *Male*  *Female* | 8 (67)  4 (33) |
| Rank  *PGY-1*  *PGY-2 PGY-3* | 4 (33)  4 (33)  4 (33) |
| USMLE Exam Scores*  Step 1  Step 2 | 239  251 |
| POCUS training before residency†  *Yes – trained in medical school*  *Yes – trained separate from medical school*  *No* | 2 (50)  0 (0)  2 (50) |
| Career plans  *Hospitalist*  *Pulmonary/Critical Care*  *Cardiology*  *Gastroenterology*  *Rheumatology*  *Hematology/Oncology*  *Endocrinology*  *Primary Care*  *Other – Infectious Diseases/Hospitalist*  *Undecided* | 2 (17)  2 (17)  2 (17)  2 (17)  1 (8)  0 (0)  0 (0)  0 (0)  1 (8)  2 (17) |

*Mean (standard deviation). †Data only available from PGY-1 residents. POCUS, point of care ultrasound; PGY, post-graduate year; USMLE, United States Medical Licensing Exam.

**Supplemental Table 3 – Frequency of Use of Diagnostic and Procedural POCUS Applications by POCUS Track Residents**

| **Diagnostic POCUS Applications** | **PGY-1**  n (%) | **PGY-2**  n (%) | **PGY-3**  n (%) |
| --- | --- | --- | --- |
| Heart (left ventricular systolic function, pericardial effusion, right heart dilation)  *Never*  *Few times per month*  *≥1 time per week* | 0 (0)  2 (50)  2 (50) | 0 (0)  1 (25)  3 (75) | 0 (0)  0 (0)  4 (100) |
| Lungs (A-lines, B-lines, consolidation, pleural effusion, pneumothorax)  *Never*  *Few times per month*  *≥1 time per week* | 0 (0)  2 (50)  2 (50) | 0 (0)  1 (25)  3 (75) | 0 (0)  1 (25)  3 (75) |
| DVT of lower extremities  *Never*  *Few times per month*  *≥1 time per week* | 2 (50)  2 (50)  0 (0) | 1 (25)  3 (75)  0 (0) | 0 (0)  3 (75)  1 (25) |
| IVC/volume assessment  *Never*  *Few times per month*  *≥1 time per week* | 1 (25)  1 (25)  2 (50) | 0 (0)  1 (25)  3 (75) | 0 (0)  0 (0)  4 (100) |
| Renal  *Never*  *Few times per month*  *≥1 time per week* | 1 (25)  2 (50)  1 (25) | 0 (0)  1 (25)  3 (75) | 0 (0)  4 (100)  0 (0) |
| Bladder  *Never*  *Few times per month*  *≥1 time per week* | 0 (0)  3 (75)  1 (25) | 0 (0)  3 (75)  1 (25) | 0 (0)  4 (100)  0 (0) |
| Biliary  *Never*  *Few times per month*  *≥1 time per week* | 2 (50)  2 (50)  0 (0) | 2 (50)  2 (50)  0 (0) | 1 (25)  3 (75)  0 (0) |
| Aorta  *Never*  *Few times per month*  *≥1 time per week* | 2 (50)  1 (25)  1 (25) | 1 (25)  3 (75)  0 (0) | 1 (25)  2 (50)  1 (25) |
| Peritoneal free fluid  *Never*  *Few times per month*  *≥1 time per week* | 0 (0)  2 (50)  2 (50) | 0 (0)  1 (25)  3 (75) | 1 (25)  0 (0)  3 (75) |
| Soft tissue  *Never*  *Few times per month*  *≥1 time per week* | 1 (25)  3 (75)  0 (0) | 0 (0)  4 (100)  0 (0) | 2 (50)  1 (25)  1 (25) |
| Musculoskeletal  *Never*  *Few times per month*  *≥1 time per week* | 3 (75)  1 (25)  0 (0) | 2 (50)  2 (50)  0 (0) | 2 (50)  2 (50)  0 (0) |
| **Procedural Applications** | | | |
| Paracentesis  *Never*  *Few times per month*  *≥1 time per week* | 0 (0)  4 (100)  0 (0) | 0 (0)  2 (50)  2 (50) | 0 (0)  3 (75)  1 (25) |
| Thoracentesis  *Never*  *Few times per month*  *≥1 time per week* | 0 (0)  4 (100)  0 (0) | 0 (0)  3 (75)  1 (25) | 0 (0)  3 (75)  1 (25) |
| Lumbar puncture  *Never*  *Few times per month*  *≥1 time per week* | 1 (25)  3 (75)  0 (0) | 2 (50)  2 (50)  0 (0) | 1 (25)  3 (75)  0 (0) |
| Central line  *Never*  *Few times per month*  *≥1 time per week* | 1 (25)  3 (75)  0 (0) | 0 (0)  3 (75)  1 (25) | 0 (0)  3 (75)  1 (25) |
| Arterial line  *Never*  *Few times per month*  *≥1 time per week* | 1 (25)  3 (75)  0 (0) | 0 (0)  3 (75)  1 (25) | 0 (0)  3 (75)  1 (25) |
| Peripheral IV  *Never*  *Few times per month*  *≥1 time per week* | 4 (100)  0 (0)  0 (0) | 2 (50)  2 (50)  0 (0) | 2 (50)  2 (50)  0 (0) |
| Arthrocentesis  *Never*  *Few times per month*  *≥1 time per week* | 4 (100)  0 (0)  0 (0) | 3 (75)  1 (25)  0 (0) | 1 (25)  3 (75)  0 (0) |

**Supplemental Table 4 – Comfort Level with Diagnostic and Procedural POCUS Applications**

| **Diagnostic POCUS Applications*** | | **PGY-1**  n (%) | | **PGY-2**  n (%) | | **PGY-3**  n (%) | |
| --- | --- | --- | --- | --- | --- | --- | --- |
| Lungs (A-lines, B-lines, consolidation, pleural effusion, pneumothorax)  *Not comfortable*  *Somewhat comfortable*  *Very comfortable* | | 0 (0)  3 (75)  1 (25) | | 0 (0)  0 (0)  4 (100) | | 0 (0)  0 (0)  4 (100) | |
| DVT of lower extremities  *Not comfortable*  *Somewhat comfortable*  *Very comfortable* | | 1 (25)  3 (75)  0 (0) | | 0 (0)  1 (25)  3 (75) | | 0 (0)  2 (50)  2 (50) | |
| IVC/volume assessment  *Not comfortable*  *Somewhat comfortable*  *Very comfortable* | | 1 (25)  3 (75)  0 (0) | | 0 (0)  0 (0)  4 (100) | | 0 (0)  0 (0)  4 (100) | |
| Renal  *Not comfortable*  *Somewhat comfortable*  *Very comfortable* | | 0 (0)  3 (75)  1 (25) | | 0 (0)  0 (0)  4 (100) | | 0 (0)  2 (50)  2 (50) | |
| Bladder  *Not comfortable*  *Somewhat comfortable*  *Very comfortable* | | 0 (0)  4 (100)  0 (0) | | 0 (0)  1 (25)  3 (75) | | 0 (0)  2 (50)  2 (50) | |
| Biliary  *Not comfortable*  *Somewhat comfortable*  *Very comfortable* | | 1 (25)  3 (75)  0 (0) | | 1 (25)  2 (50)  1 (25) | | 1 (25)  3 (75)  0 (0) | |
| Aorta  *Not comfortable*  *Somewhat comfortable*  *Very comfortable* | | 1 (25)  3 (75)  0 (0) | | 1 (25)  1 (25)  2 (50) | | 0 (0)  3 (75)  1 (25) | |
| Peritoneal free fluid  *Not comfortable*  *Somewhat comfortable*  *Very comfortable* | | 0 (0)  1 (25)  3 (75) | | 0 (0)  0 (0)  4 (100) | | 0 (0)  1 (25)  3 (75) | |
| Soft tissue  *Not comfortable*  *Somewhat comfortable*  *Very comfortable* | | 0 (0)  4 (100)  0 (0) | | 0 (0)  2 (50)  2 (50) | | 0 (0)  3 (75)  1 (25) | |
| Musculoskeletal  *Not comfortable*  *Somewhat comfortable*  *Very comfortable* | | 3 (75)  1 (25)  0 (0) | | 1 (25)  1 (25)  2 (50) | | 1 (25)  3 (75)  0 (0) | |
| **Procedural Applications** | | | | | | | |
| Paracentesis  *Not comfortable*  *Somewhat comfortable*  *Very comfortable* | | 0 (0)  2 (50)  2 (50) | | 0 (0)  0 (0)  4 (100) | | 0 (0)  0 (0)  4 (100) | |
| Thoracentesis  *Not comfortable*  *Somewhat comfortable*  *Very comfortable* | | 1 (25)  3 (75)  0 (0) | | 0 (0)  0 (0)  4 (100) | | 0 (0)  1 (25)  3 (75) | |
| Lumbar puncture  *Not comfortable*  *Somewhat comfortable*  *Very comfortable* | | 2 (50)  2 (50)  0 (0) | | 1 (25)  2 (50)  1 (25) | | 0 (0)  3 (75)  1 (25) | |
| Central line  *Not comfortable*  *Somewhat comfortable*  *Very comfortable* | | 2 (50)  2 (50)  0 (0) | | 0 (0)  1 (25)  3 (75) | | 0 (0)  1 (25)  3 (75) | |
| Arterial line  *Not comfortable*  *Somewhat comfortable*  *Very comfortable* | | 1 (25)  2 (50)  1 (25) | | 0 (0)  0 (0)  4 (100) | | 0 (0)  1 (25)  3 (75) | |
| Peripheral IV  *Not comfortable*  *Somewhat comfortable*  *Very comfortable* | | 3 (75)  1 (25)  0 (0) | | 0 (0)  4 (100)  0 (0) | | 2 (50)  1 (25)  1 (25) | |
| Arthrocentesis  *Not comfortable*  *Somewhat comfortable*  *Very comfortable* | | 4 (100)  0 (0)  0 (0) | | 3 (75)  1 (25)  0 (0) | | 0 (0)  4 (100)  0 (0) | |

*Comfort level with cardiac ultrasound was not obtained due to an error in the online survey instrument.
